# The importance of controlled mating in honeybee breeding

**DOI:** 10.1186/s12711-019-0518-y

**Published:** 2019-12-12

**Authors:** Manuel Plate, Richard Bernstein, Andreas Hoppe, Kaspar Bienefeld

**Affiliations:** Institute for Bee Research, Friedrich-Engels-Str. 32, 16540 Hohen Neuendorf, Germany

## Abstract

**Background:**

Controlled mating procedures are widely accepted as a key aspect of successful breeding in almost all animal species. In honeybees, however, controlled mating is hard to achieve. Therefore, there have been several attempts to breed honeybees using free-mated queens. In such breeding schemes, selection occurs only on the maternal path since the drone sires are random samples of the population. The success rates of breeding approaches without controlled mating have so far not been investigated on a theoretical or simulation-based level.

**Methods:**

Stochastic simulation studies were carried out to examine the chances of success in honeybee breeding with and without controlled mating. We investigated the influence of different sizes of breeding populations (500, 1000, 2000 colonies per year) and unselected passive populations (0, 500, 1000, 2000, infinitely many colonies per year) on selection for a maternally (queen) and directly (worker group) influenced trait with moderate ($r_{md}=-\,0.53$) or strong ($r_{md}=-\,0.88$) negative correlation between the two effects. The simulations described 20 years of selection.

**Results:**

Our simulations showed a reduction of breeding success between 47 and 99% if mating was not controlled. In the most drastic cases, practically no genetic gain could be generated without controlled mating. We observed that in the trade-off between selection for direct or maternal effects, the absence of mating control leads to a shift in favor of maternal effects. Moreover, we describe the implications of different breeding strategies on the unselected passive population that benefits only indirectly via the transfer of queens or drones from the breeding population. We show that genetic gain in the passive population develops parallel to that of the breeding population. However, we found a genetic lag that became significantly smaller as more breeding queens served as dams of queens in the passive population.

**Conclusions:**

We conclude that even when unwanted admixture of subspecies can be excluded in natural matings, controlled mating is imperative for successful breeding efforts. This is especially highlighted by the strong positive impact that controlled mating in the breeding population has on the unselected passive population.

## Background

The beginning of the modern era of animal breeding owes everything to Sir Robert Bakewell (1725–1795). Sir Bakewell combined strict record-keeping with the intentional mating of closely related animals for the expression of desirable traits in the population. His breeding strategies proved successful and were soon copied across Europe and North America [1–3]. An important factor in his success was the strict separation of male and female individuals except for mating, which took place under controlled conditions. Today, controlled mating is still a crucial factor in successful animal breeding. Species for which controlled mating is hard to achieve, such as aquaculture species, lag behind in breeding success partly for this reason [4, 5]. A lack of controlled mating is also a common inhibitory factor in successful animal breeding in developing countries [6, 7]. Modern breeding strategies, involving genetic evaluation, rely heavily on reliable pedigree data, further strengthening the importance of controlled mating [8].

In comparison with other agricultural species, controlled mating in the honeybee appears to be especially hard to achieve. A few days after hatching, a young honeybee queen will undertake one or several nuptial flights to drone congregation areas during which she mates in mid-air with an average of 12 drones from neighboring hives [9]. The exact number and origin of the drones cannot usually be observed [10]. The necessity of mating control became first apparent in the middle of the nineteenth century when the Italian honeybee (*Apis mellifera ligustica*) was introduced to Switzerland (1843), Germany (1853), and the United States (1860) [11, 12]. Previously, only the dark honeybee (*A. m. mellifera*) had been maintained in these areas [12, 13]. In the middle of the nineteenth century, controlled mating was not seen as a means of selective breeding but rather a means of avoiding the newly introduced subspecies to cross with the native population [13]. Strategies for controlled mating involved unsuccessful efforts of tethering the queen or enclosing the queen and drones in a tent. Attempts to achieve controlled mating by delaying the flight time of queens and drones to avoid the time window of other drones’ natural flight were more successful [11, 14, 15]. This practice, which has recently been rediscovered and became known as the Horner system, was occasionally used in Germany and the United States in the late nineteenth century [13, 15, 16]. Mating control via geographic isolation of the queen and the desired drones on so-called isolated mating stations was first (unsuccessfully) attempted by T. C. von Baldenstein in Switzerland in 1848 [17]. During the end of the nineteenth century, this technique was repeatedly applied by Swiss beekeepers, mainly under the leadership of U. Kramer from Zurich [13, 14, 18]. The concept of isolated mating stations has henceforth been developed further and is very popular in Central European honeybee breeding to this day [19]. The first reports of successful artificial inseminations of honeybee queens date back to the late nineteenth century. During the 1940s and 1950s, artificial insemination was further developed into a practical tool in economic bee breeding and is still used today [13, 14, 20].

Controlled matings allowed beekeepers to keep accurate stud books including pedigree and performance information for use in directional breeding. The systematic collection of such data was introduced in Germany around 1950 [21, 22]. In 1994, the best linear unbiased prediction (BLUP) methodology [8] was adapted to the honeybee [23] and has since yielded significant genetic improvement in all selection traits [24]. In the course of the SmartBees project [25], breeding efforts have begun in numerous European countries after the development of standardized performance testing protocols [26, 27]. To date, infrastructure for controlled mating has not been created in many European regions and its introduction will be connected with considerable logistic efforts. As most local breeders have not had any experience with controlled mating so far, it is unclear how willingly they are going to invest in these extra expenditures. Therefore the following question arises: is successful breeding possible without controlled mating by selecting dam queens only? In this context, it might be beneficial for a breeder to distribute colonies from her/his own stock among the neighboring beekeepers to increase the probability of his/her own queens mating with drones carrying good genetic material.

In this study, we compare the genetic progress in honeybee populations undergoing selection with either free mating or mating on isolated mating stations. The uncontrolled mating procedure makes it necessary to consider an unselected passive population besides the breeding population and the possible exchange of queens and drones between the populations. In addition to the genetic progress in the breeding population, this set-up also allows for further investigations on the individual contribution of maternal and direct effects under various breeding conditions. Furthermore, we examined how breeding and passive population affect each other and in particular, how the passive population can benefit from changes in the breeding population. The situation of two or more partially connected populations following different selection principles has previously been studied in other agricultural species in the context of nucleus breeding schemes both theoretically [28, 29] and by simulations [30, 31]. However, to our knowledge, there has been no such study for the honeybee with its biological peculiarities. Furthermore, none of the studies we are aware of explicitly explored the role of controlled mating in animal breeding schemes.

## Methods

We used the program BeeSim [32] to simulate the construction of honeybee populations consisting of queens and their workers as well as drones. All simulated queens belonged to one of three mutually exclusive categories:

- Breeding queens (BQ) were queens whose colonies underwent performance tests and that were subject to selection.
- Drone producing queens (DPQ) were queens that produced the drones with which BQ could mate on a mating station.
- Passive queens (PQ) formed the unselected passive population. They did not undergo any performance testing, breeding value estimation or selection procedure.

We simulated various population sizes of the breeding population and passive population. We assumed that the numbers of BQ and PQ born in each year were constant and referred to those numbers as $N_b$ and $N_p$, respectively. We considered the values $N_b = 500$, $N_b=1000$, and $N_b=2000$, as well as $N_p=0$, $N_p=500$, $N_p=1000$, $N_p=2000$, and $N_p=\infty$. In the case of controlled mating, we considered different numbers $N_s$ of mating stations. In particular, we simulated $N_s=5$, $N_s=10$, or $N_s=20$ mating stations per year, each equipped with a sister group of eight DPQ. By stating that $N_s=0$, we indicate that no controlled mating took place. Mating stations correspond with sires in other species [23, 32–34]; the relatively small values for $N_s$ are realistic due to the large logistic efforts of station maintenance.

All dams of breeding queens were necessarily breeding queens themselves. However, for the passive population we assumed that PQ could have dams from either population and considered different relative proportions *q* of PQ that had a dam from the breeding population. We simulated the different rates $q=0$, $q=0.25$, $q=0.5$, $q=0.75$, and $q=1$.

We selected for a directly (worker group) and maternally (queen) affected quantitative trait with an additive maternal genetic variance of $\sigma _{A,m}^2=1$, an additive direct variance of $\sigma _{A,d}^2=2$, and a residual variance of $\sigma _E^2=1$. We chose two different values for the correlation between the effects: one set of simulations was run with a medium negative correlation of $r_{md}=-\,0.53$ (i.e. covariance $\sigma _{A,md}=-\,0.75$) and another set with a stronger negative correlation of $r_{md}=-\,0.88$ ($\sigma _{A,md}=-\,1.25$). These numbers correspond to maternal and direct heritabilities of $h^2_m=0.53$, $h_d^2=0.37$ in the case of $r_{md}=-\,0.53$, and $h^2_m=0.72$, $h_d^2=0.46$ in the case of $r_{md}=-\,0.88$ (see [35] for a detailed description of the calculation of heritabilities for honeybees, where the direct effect reflects the mean of the worker group). The chosen numbers roughly represent those reported in the literature for parameter estimates for economically important traits such as honey yield or swarming behavior [35, 36].

All possible combinations of the parameters $N_b$, $N_p$, $N_s$, *q*, and $r_{md}$ were simulated separately over the course of 20 years and repeated 100 times in order to obtain stable results. An exception formed the parameter choices $N_p=0$ and $N_p=\infty$, which were only simulated in combination with $q= 0$, leading to a total number of 408 simulation settings (see Table 1).

**Table 1:** Overview of the parameters used for the simulations

$N_b$	$N_p$	$N_s$	*q*	$r_{md}$
500	0^a^	0^b^	0	− 0.53
1000	500	5	0.25	− 0.88
2000	1000	10	0.5	
	2000	20	0.75	
	$\infty$^a^		1	

$N_b$ number of breeding queens per year, $N_p$ number of passive queens per year, $N_s$ number of isolated mating stations, *q* relative proportion of passive queens with breeding queen dams, $r_{md}$ correlation between maternal and direct effect

^a^Only in combination with $q = 0$

^b^Indicates uncontrolled mating

The animals’ genetics were simulated for a directly and maternally influenced trait according to an infinitesimal model that accounts for the haploid nature of drones. Queens of the base population were equipped with direct and maternal true breeding values following a normal distribution with mean 0 and variance ${\varvec{\Sigma }}_A$ given by $\sigma _{A,m}^2$, $\sigma _{A,d}^2$, and $\sigma _{A,md}$. The inheritance of true breeding values from a queen *Q* to a drone *D* was realized as:

1 $$\begin{aligned} \mathrm {\mathbf {TBV}}_D=\mathrm {\mathbf {TBV}}_Q+\sqrt{1-F_Q}\cdot {\varvec{\Phi }}, \end{aligned}$$

and from a queen *Q* and drone *D* to an offspring queen *R* as:

2 $$\begin{aligned} \mathrm {\mathbf {TBV}}_R=\frac{1}{2}\left( \mathrm {\mathbf {TBV}}_D +\mathrm {\mathbf {TBV}}_Q\sqrt{1-F_Q}\cdot {\varvec{\Phi }}\right) . \end{aligned}$$

Hereby, $F_Q$ denotes the inbreeding coefficient of *Q* and ${\varvec{\Phi }}$ denotes a $N({\varvec{0}},{\varvec{\Sigma }}_A)$-distributed Mendelian sample term. Finally, worker groups obtained their breeding values as the mean value of their queen’s TBV and the average TBV of their sire drones. See [32] for a detailed description of the infinitesimal model for honeybees.

### Breeding population

When mating took place in a controlled manner, the breeding population was simulated as described in [32], i.e., each breeding queen produced a worker group and underwent performance testing. Furthermore, each year the best 20% of 2-year-old breeding queens based on a BLUP evaluation were selected as dams and each produced five breeding queens as offspring. The $N_s$ best three-year-old queens each produced a sister group as set-up for a mating station on which the newly created queens were mated with 12 drones each.

In the case of uncontrolled matings, breeding queens mated with 12 drones whose dams, one to three years old, were picked randomly from the entire (i.e. breeding and passive) population. Contrary to usual beekeeping practice, we did not simulate any culling or other exclusions of queens, so that one, two and three year old queens were equally represented among the dams of the drones. Thus, the probability of a drone in such a mating to come from a breeding colony was $p=\frac{N_b}{N_b+N_p}$. In the case of an infinite passive population, the dams of the drones were selected exclusively from the passive population. Also when matings were uncontrolled, a BLUP evaluation was performed each year and the best 20% of breeding queens were selected to produce five daughters each.

Regardless of the mating procedure, the true breeding values were passed according to Eqs. 1 and 2. However, when the inverse additive relationship matrix for the BLUP evaluations was calculated, only the information that is available in reality was taken into account. In particular, with uncontrolled mating, all sires were assumed to be unknown. The inverse additive relationship matrix was calculated with the bee specific approach of [37] following the ideas of [33], which combines the classical inversion strategy of Henderson [38] with the bee specific properties of haploid drones while considering the complex situation on mating stations.

### Passive population

After being generated, each queen of the passive population mated with 12 drones whose dams, one to three years old, were picked randomly from the entire (i.e. breeding and passive) population. Thus, the probability that a drone in such a mating comes from a breeding colony was also $p=\frac{N_b}{N_b+N_p}$, as in a breeding population without controlled mating.

When it came to generating new passive queens, a relative proportion *q* of these was created as offspring of randomly chosen BQ one to three years old. For the remaining passive queens, a dam was randomly selected among the passive queens one to three years old. The maternal generation interval for the passive queens was thus chosen to be more variable than that of the breeding population since these colonies do not have to follow the somewhat strict schedule of performance testing and subsequent selection.

The passive population was not included in the BLUP breeding value estimation and no worker groups were simulated for the passive queens. However, queens and drones from the passive population did inherit and pass on true breeding values.

#### Infinite passive population

Unlike the finite passive populations, the infinite passive population was not explicitly simulated. We assumed that, in an infinite passive population which does not receive any queens from the breeding population ($q=0$), the passive population would not undergo any genetic changes due to selection efforts in the breeding population. Thus, the passive population was only needed when breeding queens were left to mate without control. For that purpose, drones were created as if they belonged to the base population.

## Results

### Genetic progress in the breeding population

**Fig. 1:**
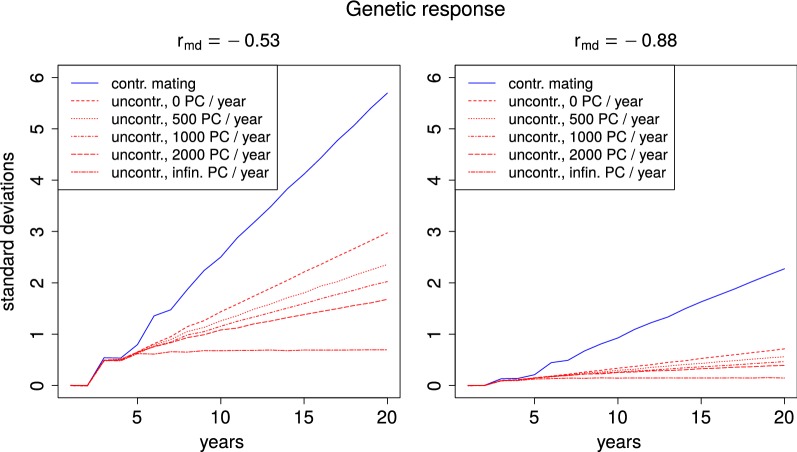
Genetic response with and without controlled mating. Genetic response to selection over the course of 20 years with a moderate ($r_{md}=-\,0.53$) and strong ($r_{md}=-\,0.88$) negative correlation between maternal and direct effects. Results are shown for a breeding population of 1000 colonies per year and various passive population sizes with (solid blue line) and without (dotted red lines) controlled mating. The passive population did not receive any dams from the breeding population ($q=0$)

We investigated how the queens’ true breeding values changed over the course of 20 years. In all simulated settings, the average true breeding value in the first two years was close to zero, since the breeding queens of these years formed the base population. In years 3 to 5, a genetic response was observed. We defined the total breeding value of a queen as the sum of her direct and maternal breeding values. In the models with a moderately negative correlation between direct and maternal effects, $r_{md}=-\,0.53$, the accumulated gain in total breeding values of queens in year 5 was between 0.63 and 0.81 units with little differences between the population sizes and breeding schemes. With a strong negative correlation, $r_{md}=-\,0.88$, a genetic response of 0.12 to 0.23 units could be accumulated by year 5. In both cases, settings with controlled mating performed slightly better. After the first five years, genetic gain increased nearly linearly in all settings. However, the rate of genetic improvement varied drastically among the different set-ups. On average, without controlled mating, the genetic gain from year 5 to year 20, measured in total breeding values of queens, was 75% lower than with controlled mating. Based on the individual setting, the range of reduction in breeding success was from 47 to 99% (see Fig. 1).

### Impact of population proportions

While all breeding schemes with uncontrolled mating performed significantly worse than their counterparts with controlled mating, the difference was more marked, when the ratio between BQ and PQ was small (see Fig. 1, Table 2). The data suggest, that, without controlled mating, breeding success depends linearly on the relative proportion *p* of BQ in the total population, i.e. $\frac{N_b}{N_b+N_p}$. The correlation between this value and the genetic gain between years 5 and 20 was 0.993 for $r_{md}=-\,0.53$ and 0.996 for $r_{md}=-\,0.88$.

**Table 2:** Genetic gain from year 5 to 20 in settings with controlled and uncontrolled mating for different correlations between direct and maternal effects ($r_{md}$) and various sizes of breeding and passive populations

	Contr. mating	Uncontrolled mating				
		0 PQ	500 PQ	1000 PQ	2000 PQ	$\infty$ PQ
$$r_{md}=-\,0.53$$						
500 BQ	4.68	2.31	1.37	1.07	0.74	0.07
1000 BQ	4.92	2.32	1.71	1.40	1.03	0.07
2000 BQ	5.08	2.34	1.98	1.72	1.40	0.08
$$r_{md}=-\,0.88$$						
500 BQ	1.79	0.56	0.32	0.24	0.19	0.02
1000 BQ	2.04	0.56	0.41	0.32	0.25	0.02
2000 BQ	2.19	0.56	0.47	0.40	0.33	0.02

Numbers for controlled mating are averages over simulation outcomes for all positive values of $N_s$

When parts of the passive queen population had dams from the breeding population ($q>0$), the genetic response without controlled mating could be slightly improved. The improvements in genetic gain due to positive values of *q* were stronger when the breeding population was small compared to the passive population: in the setting with 500 BQ and 2000 PQ the breeding success was roughly doubled from $q=0$ to $q=1$, whereas in the setting with 2000 BQ and 500 PQ, there was only an improvement of between 7 and 8%. In no case, however, could the improvement of genetic gain due to $q>0$ make up for the negative effects of the lack of controlled mating (see Table 3).

**Table 3:** Genetic gain from year 5 to 20 in in the breeding population without controlled mating when different proportions *q* of the PQ have dams from the breeding population

BQ	PQ	$r_{md}=-\,0.53$						$r_{md}=-\,0.88$					
		Uncontrolled, $q=$					Contr.	Uncontrolled, $q=$					Contr.
		0.0	0.25	0.5	0.75	1.0		0.0	0.25	0.5	0.75	1.0	
500	500	1.37	1.54	1.66	1.72	1.77	4.67	0.32	0.36	0.39	0.39	0.43	1.77
	1000	1.07	1.23	1.38	1.51	1.58	4.71	0.24	0.29	0.33	0.35	0.37	1.78
	2000	0.74	1.02	1.17	1.34	1.42	4.71	0.19	0.25	0.28	0.31	0.34	1.80
1000	500	1.71	1.82	1.88	1.93	1.97	4.93	0.41	0.43	0.44	0.46	0.47	2.02
	1000	1.40	1.54	1.65	1.73	1.78	4.92	0.32	0.36	0.38	0.41	0.42	2.03
	2000	1.03	1.24	1.38	1.51	1.60	4.96	0.25	0.29	0.33	0.36	0.38	2.02
2000	500	1.98	2.04	2.07	2.09	2.13	5.07	0.47	0.48	0.49	0.50	0.50	2.19
	1000	1.72	1.81	1.89	1.93	1.98	5.07	0.40	0.43	0.44	0.45	0.46	2.20
	2000	1.40	1.55	1.64	1.73	1.79	5.08	0.33	0.36	0.38	0.41	0.42	2.19

The corresponding rates of gain with controlled mating are given for comparison

When mating took place in a controlled manner, the realized number of mating stations had only a minor effect on the genetic gain after 20 years (see Table 4). Different numbers of mating stations caused deviations of up to 4.8% in the simulations with a moderately negative correlation between direct and maternal effects. In the simulations with a strong negative correlation, the deviations ranged up to 10.5%. There was no clear indication for an ideal number of mating stations. Traits with a stronger negative correlation ($r_{md}=-\,0.88$) tended to prefer more mating stations than traits with a moderate negative correlation ($r_{md}=-\,0.53$).

**Table 4:** Genetic gain from year 5 to 20 in different settings with controlled mating

BQ per year	$r_{md}=-\,0.53$			$r_{md}=-\,0.88$		
	$N_s=5$	$N_s=10$	$N_s=20$	$N_s=5$	$N_s=10$	$N_s=20$
500	4.70	4.61	4.40	1.78	1.90	1.89
1000	4.96	4.93	4.76	1.86	2.02	2.05
2000	5.21	5.20	5.07	1.94	2.12	2.19

### Direct and maternal effects

**Fig. 2:**
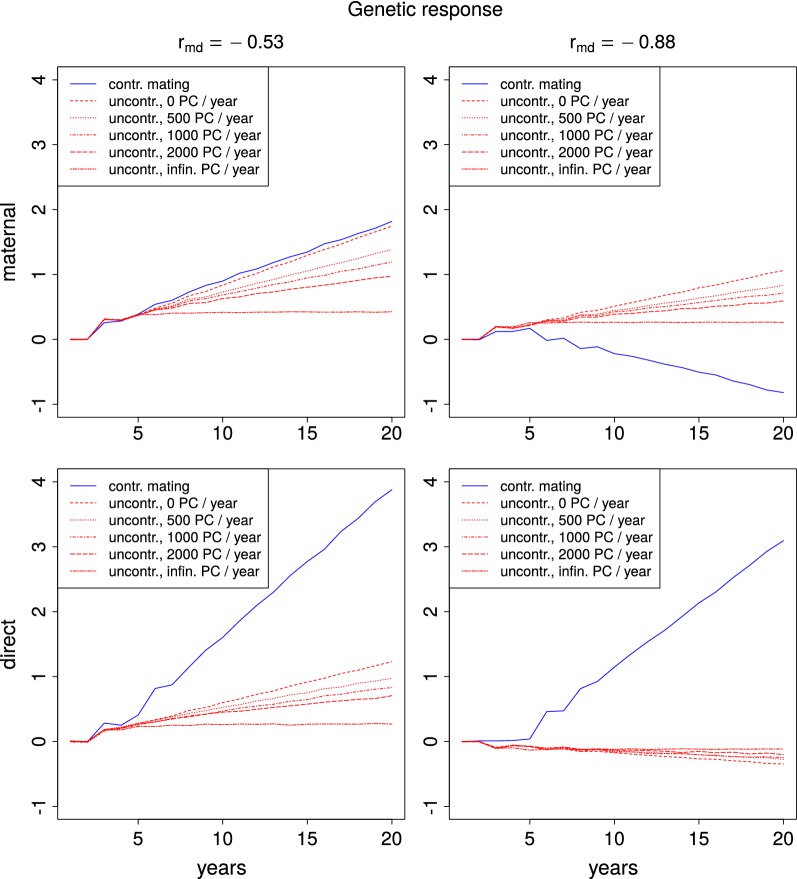
Genetic response for direct and maternal effects. Genetic response to selection of maternal and direct effects over the course of 20 years with a moderate ($r_{md}=-\,0.53$) and strong ($r_{md}=-\,0.88$) negative correlation between maternal and direct effects. Results are shown for a breeding population of 1000 colonies per year and various passive population sizes with (solid blue line) and without (dotted red lines) controlled mating. The passive population did not receive any dams from the breeding population ($q=0$)

Different simulation settings not only led to different genetic gains in total breeding values, but also had great impact on the subdivision of total genetic gain into direct and maternal genetic gain (see Fig. 2). In the following two paragraphs, we describe the progress for controlled and uncontrolled matings, separately.

#### Controlled mating

In simulations with controlled mating, the genetic gain for the direct effects was greater than for the maternal effects. When the correlation between direct and maternal effects was moderate ($r_{md}=-\,0.53$), the ratio between direct and maternal genetic gain from years 5 to 20 was between 2.01 and 2.88. Larger numbers of mating stations led to a stronger selection focus on direct effects.

A strong negative correlation ($r_{md}=-\,0.88$) even led to negative selection on maternal effects. However, the negative change in the maternal effects was outweighed by positive selection on direct effects by a factor between 2.77 and 5.07. Here, the selection on direct effects was stronger, when there were fewer mating stations.

#### Uncontrolled mating

With uncontrolled mating, the selection focus switched to the maternal effects. With a moderate negative correlation, the ratio between maternal and direct gain was between 1.29 and 1.60 without clear dependencies on population sizes or relative proportion of passive queens with breeding queen dams (*q*).

A strong negative correlation ($r_{md}=-\,0.88$) yielded a slightly negative selection on direct effects, which was outweighed by the gain in the maternal effects by a factor between 2.46 and 3.39. Again, no clear dependencies could be detected.

### Changes in the passive population

**Fig. 3:**
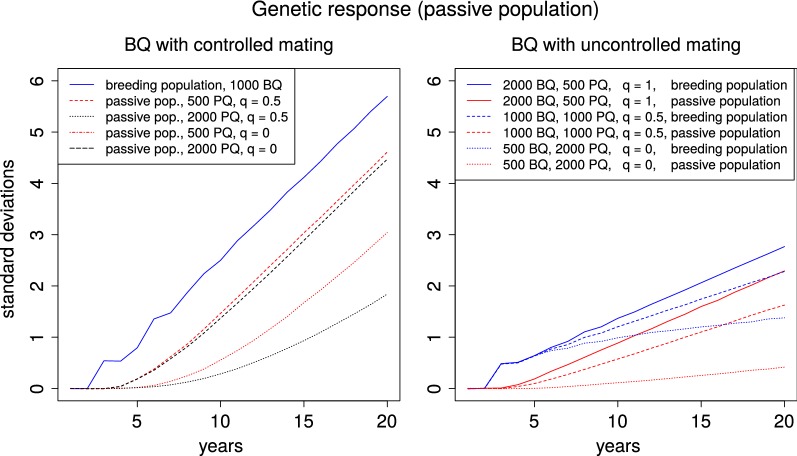
Genetic response in the passive population. Genetic response to selection in the breeding and passive populations over the course of 20 years with a moderate ($r_{md}=-\,0.53$) negative correlation between maternal and direct effects. Results are shown for a breeding population with (left hand side) or without (right hand side) controlled mating, different population sizes and different relative proportions of passive queens with breeding queen dams (*q*)

All finite passive populations showed a positive genetic response which was delayed in time compared to the breeding population. When breeding queens served as dams for at least half of the passive queens ($q\ge 0.5$), the genetic difference between breeding and passive population remained constant after a few years (see Fig. 3). For $q=1$ and controlled mating conditions in the breeding population, the passive population stayed between 1.47 and 2.03 years behind the breeding population in terms of genetic gain. The time delay was shorter for smaller breeding populations. When *q* was reduced to 0.5, it took the passive population between 3.17 and 4.24 years to reach the level of the breeding population. Here, smaller passive populations led to a smaller gap between the breeding values of the respective populations. Under uncontrolled mating conditions for the breeding population, the time difference in genetic gain increased to between 3.26 and 5.16 years for $q=1$, respectively to between 4.56 and 8.67 years for $q=0.5$. For values of $q<0.5$ and in particular for $q=0$, i.e. no breeding queen dams for the passive population, the genetic gain of the passive population was slower than that of the breeding population throughout the entire 20 years. However, in these cases the genetic response in the passive population was superlinear over the entire 20 years. Thus, it is expected that the passive population will also reach the rates of genetic gain of the breeding population, but only well after 20 years.

## Discussion

### Model choice

#### Genetic model and simulated time

Simulation studies in animal breeding mostly rely on either Fisher’s infinitesimal model [39, 40] or on finite locus models [41]. Previously, we have shown in the context of honeybee breeding that long-term simulation studies based on finite locus models are more reliable than those that use the infinitesimal model [32]. However, in the same study, we also showed that for studies that do not exceed the timeframe of 20 years, either model works equally well. Thus, we decided to use the infinitesimal model because it has fewer levels of freedom and therefore needs fewer repetitions of the simulations to obtain stable results.

The simulated time of 20 years, i.e. 10 maternal generations, is rather short for an investigation of strategies in animal breeding [42, 43]. However, such limited time frames are not without examples [44, 45] and may even be long considering the objectives of individual breeders.

In the long term, breeding schemes with uncontrolled mating will generate lower rates of inbreeding and thus a reduced loss of genetic variance. However, promising mating schemes in animal breeding that aim at avoiding high inbreeding rates, such as optimum contribution selection, generally do not compromise genetic gain to a large extent [43, 46]. Based on our current findings, it is already clear that this is not the case for uncontrolled mating of honeybees.

**Fig. 4:**
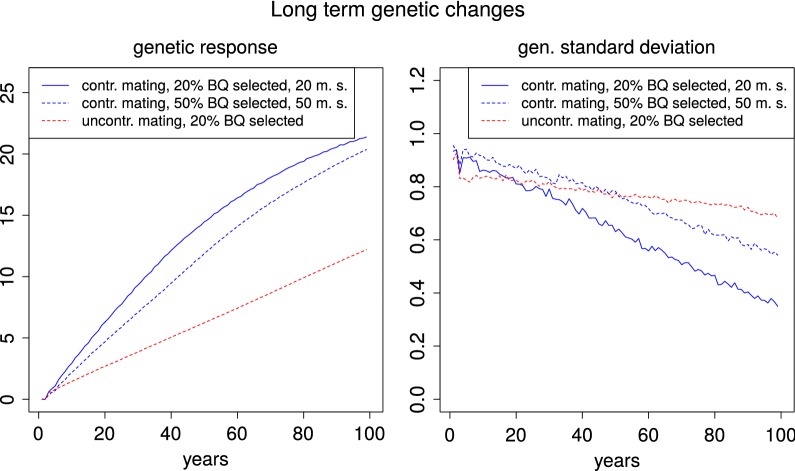
Genetic change over 100 years. Genetic response to selection (left hand side) and evolution of genetic standard deviation (right hand side) over the course of 100 years with a moderate ($r_{md}=-\,0.53$) negative correlation between maternal and direct effects. Results are shown for a breeding population of 500 colonies and a passive population of 1000 colonies per year with (blue lines) and without (dotted red line) controlled mating. Controlled mating was performed with two different selection intensities: 20 mating stations and the best 20% of BQ selected as dams (solid blue line) of 50 mating stations and the best 50% of BQ selected as dams. We assumed $q=0.5$

In order to hint at the long-term effects of controlled and uncontrolled mating, we conducted a small-scale simulation with 20 repetitions over the course of 100 years. (see Fig. 4) In this simulation, we chose the parameters $N_b=500$, $N_p=1000$, $r_{md}=-\,0.53$, and $q=0.5$ and uncontrolled mating ($N_s=0$) or controlled mating on $N_s=20$ mating stations. As a genetic model, we chose a finite locus model with 400 unlinked loci as is described in [32]. After 100 years, the genetic response in the simulation without controlled mating was reduced by 43% in comparison to the controlled mating scheme. Without controlled mating, the initial standard deviation was reduced by only 24% (63% with controlled mating). In another simulation, we reduced the loss of genetic standard deviation under controlled mating by increasing the number of mating stations to 50 and selecting 50% of all breeding queens as dams (as opposed to 20% in the rest of our simulations). By this means, we could decrease the loss of genetic standard deviation from 63 to 43% while the genetic response after 100 years decreased by only 5%. We expect that an implementation of more sophisticated selection strategies, such as optimum contribution selection [46], can yield high response rates with even smaller reductions of variance. Thus, in our opinion renouncing controlled mating is a clearly inferior breeding practice for honeybees, also in the long term.

#### Natural selection

In the honeybee, resistance to the parasitic *Varroa* mite is a trait that is often regarded to be a fitness advantage [47, 48] and some breeding strategies rely on the assumption that drones from resistant colonies are more likely to reproduce than those from susceptible colonies [19, 49]. Other commercial quantitative traits in the honeybee, such as honey production or gentleness, appear to have negligible implications on colony fitness.

In our studies, we assumed an absence of natural selection against or in favor of the simulated trait and thus that our simulations accurately describe the situation for most commercial traits. For the *Varroa* resistance trait, the absence of controlled mating may be less inferior than our simulations suggest since in uncontrolled matings resistant drones also have a fitness advantage over susceptible drones. In small populations, the so-called tolerance mating stations as they are described in [49, 50] may be a promising alternative to classical mating stations for this trait.

#### Age structure

In our simulations, all queens between one and three years old had the same chance of being the dam of a drone involved in an uncontrolled mating, and the number of queens was the same in each age group. Similarly, the dams of passive queens were chosen randomly from queens one to three years old. In reality, there will be more younger queens because several queens will not reach three years of age due to illnesses, colony losses or requeening practice [26, 51]. Furthermore, it is likely that in reality, the queen-to-queen generation interval in the passive population will be larger than in the breeding population because in the passive population there is no urge to rapidly improve the genetic material. This effect will become even stronger in the future due to the possibilities of shorter generation intervals due to genomic selection [34, 52]. However, this study aimed at investigating the implications of controlled mating on breeding success. In order to be able to quantify its effects, we avoided intermingling them with other factors such as age structures or generation intervals. The fact that queens of all ages are equally represented leads to an average queen-to-queen generation interval in the passive population of two years, which equals the queen-to-queen interval in the breeding population. This facilitates the comparison between the breeding and passive populations.

Simulation studies with realistic age structures and generation intervals would require a detailed knowledge of the behavior of beekeepers of the passive population. However, due to the nature of the passive population, no such data is available. In areas where beekeeping is mainly carried out by large commercial operators, procedures are likely to be highly standardized. However, the diverse structure of beekeeping in Europe with many recreational beekeepers suggests a wide variety in individual practices. Nevertheless, we expect that realistic simulations would lead to results that are similar to those presented here.

#### Total breeding value

In accordance with most of the literature on bee breeding, the sum of the direct and maternal breeding values of a worker group served as the selection criterion in the present simulations. This value is generally chosen since it represents the expected genetic properties of an unmated queen offspring of the colony [23, 33, 53].

However after simulation, when we investigated the change in the genetic response in the population, we chose the sum of the direct and maternal breeding values of a queen as the total breeding value. This choice has the advantage that it allows comparing genetic progress between breeding and passive populations even though PQ are generally simulated without worker groups. Based on our previous work, [32], the selection criterion and the total breeding value as defined here generally show the same behavior. The present definition of total breeding values has previously been used in [54].

### Impact of controlled mating

The linear genetic progress is in line with other breeding simulations in the literature [32, 55]. Furthermore, the rate of genetic gain in a trait with a moderate negative correlation between direct and maternal effects ($r_{md}=-\,0.53$) under controlled mating conditions is similar to the results in [32].

To our knowledge, these are the first simulation studies on breeding strategies without mating control. We found that the absence of controlled mating clearly impaired genetic progress in all settings in two ways.

- First, it allows only for inaccurate calculations of relationships and therefore leads to a less reliable BLUP-based breeding value estimation [33].
- Second, it does not allow for selection on the paternal path and the genetic progress will continuously be hampered by queens that mate with genetically inferior drones from the breeding or passive population.

Since the drones in the passive population can not or only indirectly benefit from the breeding efforts, the risk of queens mating with undesired genetic material increases with the relative proportion of PQ in the population. The high correlation rates between the relative proportion of BQ in the population and the rate of genetic gain indicate that the impact of genetically inferior drones is the prevalent factor.

If breeders give away virgin queens to the passive population ($q>0$), this improves the average genetics of the passive population. However, this affects the breeding population only whenever a BQ mates with a drone from the passive population. This explains, why the positive influence of a positive *q* value was especially high, when the passive population was relatively large. The improvements in the breeding population due to $q>0$ were generally small, which has its reason in the indirect nature of the effect.

The situation of $q> 0$ can be compared to nucleus breeding programs in other agricultural species, where nucleus-born individuals are disseminated to the base population. In a related setting (albeit with controlled matings with sires of the base population), James [28] also derived a small positive influence of such practices.

#### An extreme case: infinite passive population

In the case of uncontrolled mating with an infinite passive population that did not receive queens from the breeding population ($q=0$), there was little extra genetic gain after a few years (see Fig. 1, Table 2). This may seem surprising at first sight as one might think that the selection of superior dam queens would have to lead to at least some improvement. However, there is a theoretical explanation to this effect as described below.

Let $\overline{\mathrm {TBV}}_t$ be the average breeding value of the breeding population in year *t*. Then, since we assumed an absence of selection in the passive population and that it cannot benefit from the breeding population ($q = 0$, $p =\frac{N_b}{N_b+\infty }=0$), the average breeding value of the infinite passive population remains constant $\overline{\mathrm {TBV}}_0$. Furthermore, we assumed that the average breeding value of the selected breeding queens in year *t* is $\overline{\mathrm {TBV}}_t+S_t$ and that $S_t\le S$ is bounded for all years. Now, once the average breeding value of the breeding population has improved by this upper bound *S*,

$$\begin{aligned} \overline{\mathrm {TBV}}_{t_0}=\overline{\mathrm {TBV}}_{0}+S, \end{aligned}$$

we obtain, for the next generation, the average of the selected queens of year $t_0$ and the drones from the passive population. I.e.,

$$\begin{aligned} \overline{\mathrm {TBV}}_{t_0+\text {generation interval}}&=\frac{1}{2}\left( \overline{\mathrm {TBV}}_{t_0}+S_{t_0}+\overline{\mathrm {TBV}}_0\right) \\&\le \frac{1}{2}\left( \overline{\mathrm {TBV}}_{0}+S+S+\overline{\mathrm {TBV}}_0\right) \\&=\overline{\mathrm {TBV}}_{0}+S \end{aligned}$$

Thus, the average breeding value of the breeding population will never exceed the maximum superiority *S* of selected dam queens.

#### Breeding with uncontrolled mating in reality

Several honeybee breeding experiments without controlled mating have been performed to improve the hygienic behavior of workers but the results of these studies are ambiguous. While [56] found only small improvements over five generations, other studies have shown short-term breeding success without controlled mating in the selection for hygienic behavior [57–59]. but did not investigate if the initial rate of genetic improvement after one generation could be maintained over longer periods of time. In fact, the results of [57] show an initial improvement in the first two years but stagnation afterwards. Like [59], our simulation studies indicated an initial breeding success under uncontrolled conditions that was only slightly inferior to selection with controlled mating. However, our simulations show that this initial genetic improvement in the first few years cannot be held up in the middle and long term.

#### Further aspects of controlled mating

In this study, we did not assume that genetic transfer between the breeding and passive populations has any implications beyond the influence on an unspecified trait. This would be the case if breeding and passive populations are genetically similar, i.e., belong to the same subspecies. In practice, however, many of the newly established bee breeding programs in Europe are confronted with the situation that the native population is heavily endangered by admixture due to the introduction of foreign honeybee subspecies [60, 61]. Besides the moral aspect of conserving native subspecies, there are also economical reasons to prevent admixture, as it has been observed that hybrids show increased aggressive behavior and native subspecies generally have fitness advantages due to local adaptation [62]. In areas, where there is a risk of crossing between subspecies, controlled mating is crucial beyond reasons of breeding progress [63, 64].

In regions that do not provide the necessary geographical features for secure mating stations, artificial insemination can be a practicable alternative [65]. Furthermore, alternative strategies for controlled mating, using time shifts in the nuptial flights, have shown promising results [16, 66].

### Direct and maternal effects

The preferred selection for direct effects under controlled mating conditions is in line with the results of [32] and can be explained by the larger direct additive genetic variance. Negative selection on maternal effects when they are strongly negatively correlated with the direct effects has been shown in simulation studies for other agricultural species [67, 68]. The role of the number of mating stations, which corresponds to the number of sires in other species, can be explained as follows. On the one hand, a small number of mating stations implies a strong selection on the paternal side, which will influence the direct breeding values of the tested worker groups positively. On the other hand, a larger number of mating stations leads to higher genetic diversity in the sires and thus in the direct effects of the worker groups. This increases the accuracy of the estimation of direct breeding values [69]. Traits with a low negative correlation between direct and maternal effects generally have higher total heritabilities and therefore more accurate breeding values and can thus benefit from an intense selection scheme. In comparison, traits for which direct and maternal effects are strongly negatively correlated need a larger number of sires for an accurate estimation of breeding values.

While maternal effects are expressed directly in the BQ that are to be selected, their direct breeding values can only be deduced via relationship information to their worker groups. This relationship information is far less accurate when uncontrolled mating is applied, which explains the stronger focus on maternal effects. This means, that a part of the reduced genetic gain in selection schemes without controlled mating is also caused by the fact that the direct breeding values cannot be assessed accurately. Therefore, the selection focus is shifted from the ideal mixture of direct and maternal effects and concentrates too strongly on the maternal effects.

The genetic progress in the breeding schemes with controlled mating indicate that with a strong negative correlation between direct and maternal effects it may be ideal to sacrifice the maternal effects in order to overcompensate the maternal genetic loss with gains in the direct effect. When the negative correlation is lower, a positive selection on both effects appears favorable. Future research on how an ideal weighting of the loci under selection on direct and maternal effects depends on their (co-)variances is of great interest.

A negative selection on maternal effects may lead to practical difficulties, even when it is overcompensated by direct genetic gain. It makes the queen more dependent on her own workers which may lead to problems in the practice of queen replacement [70, 71]. However, in practical breeding programs, no negative change of either effect has been observed so far [24, 72].

The genetic parameters in our simulations may appear somewhat extreme. In particular, a negative correlation between effects of $r_{md}=-\,0.88$ may be seen as too strong and heritabilities of the considered traits are high. We decided to use these parameters because they reflect the estimates that were obtained for economically relevant traits such as honey production or swarming behavior [21, 35]. Negative genetic correlations between direct and maternal effects have repeatedly been estimated for other farm animals and, in some cases, reached or exceeded values around − 0.9 [73, 74]. Estimation of parameters for honeybees is particularly difficult because each queen has only one worker group as offspring that can be used to separate direct effects from maternal effects. In a study on sheep, Maniatis and Pollott showed, that unreasonably strong negative correlations between effects can be estimated when the number of offspring per dam is small and performance data are missing [75]. However, honeybee simulation studies have shown that genetic parameters for honeybees can be estimated without bias [33].

In [32], honeybee breeding simulations with a weaker genetic correlation of $r_{md} = -\,0.18$ were implemented and showed only quantitative rather than qualitative differences to those with $r_{md}=-\,0.53$. Thus, we believe that the key results of the present work also hold true if the real correlation between direct and maternal effects is lower than we assumed. In addition, it is most likely that although the breeding values will be estimated with the wrong parameters it will not have a big impact [76].

Traits with indirect genetic effects often show particularly high heritabilities which are caused by the negative correlation between effects. In extreme cases, the genetic variance for individual effects may exceed the phenotypic variance, leading to heritabilities higher than 1, which are impossible in classical theory without indirect effects [77]. In honeybees, this effect is further strengthened by the fact that the direct effect is shown in a collective rather than a single individual, which causes further reduction of the phenotypic variance [35]. Therefore, it is recommended to be cautious when deriving implications from high heritabilities in honeybee traits. For example, Brascamp et al. [53] have reported that despite high heritabilities, the selection differentials in honeybee breeding schemes can be seen as low.

### Genetic progress in the passive population

To our knowledge, these are the first simulations that investigate the influence of breeding programs on the surrounding unselected population in any agricultural species. However, nucleus breeding programs with interdependent populations have been studied. In these breeding programs, an eventually parallel genetic contribution in the populations has been predicted theoretically [28] and observed in simulations [31]. Therefore, the parallel genetic progress of breeding and passive populations that was observed for $q>0$ can also be assumed in the case of a maternally self-sufficient passive population when a timeframe of more than 20 years is taken into account. It points out that decisions for the breeding population may have severe consequences for the entire population since they inevitably influence the genetic changes in the passive population with a delay in time. It clearly marks the importance of breeders who use controlled mating, since they will pave the way for the genetic improvement of the entire population. The results indicate that it is advantageous for beekeepers without breeding ambitions to obtain their queens from active breeders because it lets them benefit from the breeding activities with a shorter time delay.

## Conclusion

Our simulation study shows that controlled mating is crucial to generate genetic response over several generations. Especially in regions where breeders form a minority among the beekeepers, as it is mostly the case, controlled mating is absolutely mandatory. Moreover, depending on the exchange rates of queens and drones, the passive population can also benefit greatly from a controlled mating of BQ. Thus, applying controlled mating does not only mean a personal advantage for individual breeders but is also important for the genetic progress of the passive population.

## Data Availability

The datasets used and/or analysed during the current study are available from the corresponding author on reasonable request. The source code of the simulation program BeeSim is available at https://doi.org/10.5061/dryad.1nh544n.
